# Comparative Performance of Four Staging Classifications to Select «High-Risk» Head and Neck Cutaneous Squamous Cell Carcinomas

**DOI:** 10.3390/jcm12123929

**Published:** 2023-06-08

**Authors:** Roxane Elaldi, Emmanuel Chamorey, Renaud Schiappa, Anne Sudaka, Fabienne Anjuère, Agathe Villarmé, Dorian Culié, Alexandre Bozec, Henri Montaudié, Gilles Poissonnet

**Affiliations:** 1 Face and Neck Surgery Department, Antoine Lacassagne Center, 06100 Nice, France; 2 CNRS UMR7275, Institut de Pharmacologie Moléculaire et Cellulaire, Université Côte d’Azur, 06560 Valbonne, France; 3 Statistics Department, Antoine Lacassagne Center, 06100 Nice, Francerenaud.schiappa@nice.unicancer.fr (R.S.); 4 Anatomopathology Department, Antoine Lacassagne Center, 06100 Nice, France; 5 Dermatology Department, University Hospital of Nice, 06200 Nice, France; 6 INSERM U1065, Centre Méditerranéen de Médecine Moléculaire, Université Côte d’Azur, 06200 Nice, France

**Keywords:** cutaneous squamous cell carcinoma, head and neck, prognosis, classification system, oncology

## Abstract

Background: Many classifications exist to select patients with “high-risk” head and neck cutaneous squamous cell carcinoma (HNCSCC). Objective: To compare the performance of the Brigham and Women’s Hospital (BWH) classification with the performance of the American Joint Committee on Cancer 8th Edition (AJCC8), the Union for International Cancer Control 8th Edition (UICC8), and the National Comprehensive Cancer Network (NCCN) classifications. Methods: In this single-center retrospective study, HNCSCC resected in a tertiary care center were classified as “low-risk” or “high-risk” tumors according to the four classifications. Rates of local recurrence (LR), lymph node recurrence (NR), and disease-specific death (DSD) were collected. The performance of each classification was then calculated in terms of homogeneity, monotonicity, and discrimination and compared. Results: Two hundred and seventeen HNCSCC from 160 patients, with a mean age of 80 years, were included. For predicting the risk of any poor outcome and risk of NR, the BWH classification had the best specificity and positive predictive value. However, its concordance index was not significantly higher than that of the AJCC8 and UICC8 classifications. The NCCN classification was the least discriminant. Conclusions and Relevance: This study suggests that the BWH classification is the most appropriate for predicting the risk of poor outcomes in patients with HNCSCC when compared with the NCCN, UICC8, and AJCC8 classifications.

## 1. Introduction

Cutaneous squamous cell carcinomas (cSCC) are epithelial tumors that account for 20–50% of skin tumors. With exposure to UV radiation as the main risk factor, these tumors are mostly found on the face and neck [1]. The gold standard treatment is excisional surgery for operable patients [2], more or less followed by adjuvant radiotherapy depending on clinical and histological characteristics [2]. Lymph node dissection is indicated in patients with clinical or radiological lymph node involvement. Performance of adjuvant radiotherapy is considered in cSCC of the head and neck (HNCSCC) with positive margins after surgery, without the possibility of re-excision, or with regional nodal metastasis and extracapsular extension. Although tumors with lymph node or distant metastases involvement have a poor prognosis [3,4], local tumors have a good prognosis with more than 96% specific survival at 5 years, 4.6% local recurrence (LR), and 3.7% lymph node recurrence (NR) after excision surgery [5]. However, these results do not reflect the singularity of certain local cSCCs, particularly those located in the face and neck, more lymphophilic than cSCC from other locations, with certain subgroups of patients with a “high-risk” tumor having more than 20% LR at 2 years [6]. There are several prognostic classifications aimed at identifying “high-risk” patients and the most widely used are the American Joint Committee on Cancer 8th edition (AJCC8) [7], the Union for International Cancer Control 8^th^ edition (UICC8) [8], the Brigham and Women’s Hospital (BWH) [9], and the National Comprehensive Cancer Network 2020 (NCCN) [10] classifications. Of these classifications, only the AJCC8 classification targets only HNCSCC.

According to the most recent recommendations and publications [10,11,12], these “high-risk” patients should be offered appropriate management not only in terms of extension assessment (initial nodal imaging) but also in terms of treatment (performance of MOHS micrographic surgery) and follow-up (performance of lymph node ultrasound during follow-up). The several definitions of “high-risk” tumors are indeed responsible for heterogeneity in the therapeutic management of patients. They also increase the difficulty of patient selection in clinical trials aimed at evaluating the contribution of specific therapeutic treatments for these patients, such as sentinel node or adjuvant radiotherapy.

Authors have compared the performance of the BWH and AJCC8 classifications [6,13]. Ruiz et al., showed in a series of 680 head and neck cSCCs (HNCSCC) that the BWH classification predicted the risk of NR and disease-specific death (DSD) better than the AJCC8 classification [6], but no study has yet compared the BWH classification to the IUCC8 and NCCN classifications. 

Therefore, the objective of our study was to compare the performance of the BWH classification to the AJCC8, UICC8, and NCCN classifications, to better select “high-risk” HNCSCC, to guide the clinician in current practice, and to facilitate the construction of clinical trials. 

## 2. Methods

### 2.1. Study Design and Patient Eligibility

In this single-center retrospective study, patients with invasive HNCSCC who underwent surgery for their primary tumor or local recurrence in a tertiary care center from 2006 to 2018 were selected. HNCSCC was treated by surgical resection (MOHS micrographic surgery technique or wide excision with margins adapted to the tumor presentation). Only patients with a tumor whose surgical resection was complete (R0 status) were selected. Patients with lymph node involvement (clinical or radiological) were excluded. Performance of lymph node dissection or sentinel lymph node biopsy was also an exclusion criterion. Adjuvant radiotherapy was then performed on a few patients following a decision by the multidisciplinary consultation committee. Finally, the patients were followed up alternatively by the surgeon and dermatologist. They underwent clinical examinations every 6 months for a minimum of 2 years. Additionally, neck ultrasounds were performed as decided by the physician or if there were suspicions of NR. Subsequently, the patients continued their follow-ups with the dermatologist at least once a year for a minimum of 5 years. This retrospective study was approved by the institutional ethics committee of the Centre Antoine Lacassagne (Nice) and was conducted in compliance with the MR-004 methodology (Deliberation No. 2018-155 of 3 May 2018) and the Declaration of Helsinki. The number granted by the Health Data Hub is N° F20201109183306. In accordance with General Data Protection Regulation, all the patients were informed that their data would be used for this project, and none refused. 

### 2.2. Data Collection 

The following data were collected in the computerized patient records: sex, age, immunosuppression, tumor location, type of surgery performed (wide excision or MOHS micrographic surgery), tumor diameter, tumor differentiation, tumor invasion >6 mm (as measured from the granular layer of the adjacent normal epidermis to the base of the tumor), presence of histological and clinical perineural invasion, tumor depth, and tumor stage according to the four prognostic classifications of UICC8, AJCC8, BWH, and NCCN. Ensuing outcomes occurring during follow-ups were also recorded: LR, NR, and DSD. Patients with Human Immunodeficiency Virus (HIV), myeloproliferative or myelodysplastic diseases, cancer treated with chemotherapy, inflammatory disease treated with immunosuppressive or immunomodulatory therapies, and patients who have had an organ transplant were considered immunocompromised. The stratification of tumors according to the four classifications was deduced from the information provided by the pathology report. For locations on the eyelid, the dedicated UICC8 edition classification was used. Tumors staged as T1 and T2 in the UICC8 and AJCC8 classifications, and T1 and T2a in the BWH classification were considered “low-risk”, and tumors staged as T3, T4a, and T4b in the UICC8 and AJCC8 classifications and T2b and T3 in the BWH classification were considered “high-risk” (Table 1). LR was defined as the recurrence of a lesion in place of a previously resected lesion with an R0 resection quality. NR was defined as the appearance of lymph node invasion in the lymphatic drainage area of an N0 lesion previously resected with R0 resection quality. DSD was considered to be attributable to HNCSCC if it was due to local disease, lymph node involvement, or distant involvement or if it occurred as a result of post-operative complications or following radiotherapy or chemotherapy. The duration of follow-up was defined as the time between the date of surgery and the date of the last news.

### 2.3. Statistical Analysis

Cox proportional hazards regression was performed to calculate the risks of LR, NR, and DSD for tumors classified as “high-risk” compared to those classified as “low-risk” based on each classification. These risks are presented as hazard ratios (HR) with their 95% confidence interval (95% CI). The performance of the four classification systems, BWH, AJCC8, UICC8, and NCCN, was evaluated in terms of homogeneity (the ability of the results to be similar within the risk class to which the tumor belongs), monotonicity (the ability of the results to worsen with increasing risk class to which the tumor belongs), and discrimination (the ability of the results to differ between risk classes). To compare homogeneity, the proportions of poor outcomes (LR, NR, and DSD) in the “low-risk” tumor classes of the BWH classification were compared with those of the other classifications using the McNemar test. To compare monotonicity, the proportions of poor outcomes in the BWH “high-risk” tumor classes were compared with those in other classifications using the McNemar test. For the discrimination study, the sensitivity, specificity, positive predictive value (PPV), negative predictive value (NPV), and concordance index (C-index) of classifications to predict LR, NR, or DSD (considered as a single risk), and to predict NR only, were calculated for each classification. The sensitivity and specificity of the BWH classification were compared to those of the other classifications using the McNemar test. The C-index, which mathematically corresponds to the area under the curve (AUC) for binary results, was then calculated for each classification, and the C-index of the BWH classification was compared to the C-index of the other classifications using the Delong test. All reported *p*-values were 2-sided with type I error (α < 0.05) considered to be statistically significant. Statistical analyses were performed using R 3.6.1.

## 3. Results 

### 3.1. Population 

A total of 160 patients with 217 tumors were included in this study (135 men and 25 women). Out of the 217 tumors, 192 were primary, and 25 were recurrences. The mean age of the patients at surgery was 80 years ranging from 40 to 100 years. In total, 40/217 (18%) tumors were managed in an immunosuppressive context. Adjuvant radiotherapy was performed in one patient because of gross bone invasion. Tumor characteristics are described in Table 2. The median follow-up after the end of treatment was 20 months (Inter Quartile Range: 7–48). According to the BWH classification, 25% of tumors were at “high-risk” compared to 46% and 41% of tumors according to the AJCC8 and UICC8 classifications, respectively, and 97% of tumors according to the NCCN classification. 

### 3.2. Risk of Poor Outcomes 

In our cohort, LR occurred in 21 patients, NR occurred in 11 patients, and DSD occurred in 9 patients. The risks of LR, NR, or DSD for tumors classified as “high-risk” by each classification are outlined in Table 3. Tumors classified as “high-risk” by the BWH, AJCC8, and UICC8 classifications were significantly more at risk of developing LR or NR than “low-risk” tumors. The HR associated with the risk of the NCCN “high-risk” classification to develop poor outcomes could not be evaluated because the numbers of “low-risk” and “high-risk” tumors according to this classification were not comparable. 

### 3.3. Homogeneity and Monotonicity

There was no significant difference in homogeneity (proportion of LR, NR, and DS in “low-risk” tumors) and monotonicity (proportion of LR, NR, and DSD in “high-risk” tumors) in the BWH classification compared to the AJCC8 and UICC8 classifications. However, the NCCN classification had better homogeneity and monotonicity than the BWH classification for LR (*p* = 0.008, Table 4).

### 3.4. Discrimination

Table 5 shows the discrimination capabilities of the BWH, AJCC8, UICC8, and NCCN classifications to predict poor outcomes (LR, NR, and DSD combined as a single risk) and to predict NR. 

To predict poor outcomes (LR, NR, and DSD combined as a single risk), the BWH classification had significantly lower sensitivity than the AJCC8, UICC8, and NCCN classifications (*p* = 0.01, *p* = 0.05, and *p* = 0.001, respectively), but better specificity (*p* < 0.001 for all three classifications). The PPV of the BWH classification was the highest (PPV = 30%), while its NPV, although high (NPV = 92%), was the lowest. The BWH classification was significantly more discriminating than the NCCN classification (C-index BWH = 0.68 vs. C-index NCCN = 0.52, *p* = 0.002), but no difference was found compared with other classifications. 

To predict NR, the sensitivities of the AJCC8, UICC8, and NCCN classifications were not significantly different from the BWH classification, but the BWH classification had better specificity (*p* < 0.001 for all three classifications). Finally, the PPV of the BWH classification was higher (15%), and its NPV remained high (98%), although not higher than the other classifications. The BWH classification was significantly more discriminating than the NCCN classification in predicting the risk of NR (C-index BWH = 0.70 vs. C-index NCCN = 0.52, *p* = 0.02), but no difference was found compared with other classifications.

## 4. Discussion

Patients with HNCSCC who experience recurrence, especially in the lymph nodes, have a poor prognosis. It is, therefore, important to be able to identify “high-risk” patients in order to provide them with intensified treatment and follow-up care. Current guidelines for cSCC management recommend specific advanced approaches for “high-risk” patients, including the performance of initial nodal imaging, MOHS micrographic surgery, and intensified follow-up with nodal imaging [2,10,14,15]. Similarly, the identification of “high-risk” patients is necessary to optimize the inclusion of patients in clinical trials evaluating the benefits of sentinel lymph node biopsy, adjuvant radiotherapy, or adjuvant immunotherapy [16]. These interventions may have side effects that impact functional prognosis, so it is crucial to carefully select the subgroup of patients who are likely to benefit from them without overtreating and causing morbidity. Furthermore, the selection of these patients is even more important in patients with HNCSCC, which are more lymphophilic, with a higher risk of nodal recurrence compared to other locations [10].

After comparing the performance of the four classifications, our results suggested that the NCCN classification was significantly the least discriminative in predicting the risk of NR and poor outcomes (LR, NR, and DSD combined as a single risk). Among the other classifications, the BWH classification had a significantly higher specificity and PPV in predicting the risk of NR and poor outcomes (LR, NR, and DSD combined as a single risk) than the AJCC8 classification, which is consistent with the findings of Ruiz et al. [6] and Roscher et al. [13]. Our study also demonstrated that these results were similar when the BWH classification was compared with the UICC8 classification. However, our results showed that there was no significant difference in terms of discrimination between the BWH classification and the AJCC8 and UICC8 classifications, whereas Ruiz et al. found that the BWH classification was significantly more discriminative than the AJCC8 classification in predicting the risk of NR. This difference in results probably springs from the low power of our study. 

In addition to its lack of power, our study was a single-center study, and the tumor slides were not read by a pathologist specifically for this study. Indeed, the identification of histological risk factors, such as the estimation of the grade of differentiation of the tumor, may vary depending on the pathologist [17]. However, this lack of inter-individual reproducibility is a major limitation of the BWH classification and may modify the selection of “high-risk” patients. Furthermore, in this study, the majority of HNCSCC were not treated using the MOHS micrographic surgery [18]. Therefore, some tumors may have relapsed not because they were “high-risk” tumors but because of unclear margins not analyzed when a wide excision technique was performed. MOHS micrographic surgery is indicated for “high-risk” HNCSCC, but in most cases, a biopsy is not sufficient to classify tumors as “high-risk” using these classifications. According to the classifications studied here, their “high-risk” nature is revealed after surgical resection, which does not allow MOHS micrographic surgery to be performed. Additionally, the classifications studied here do not include certain clinical or histological criteria previously shown to be associated with a worse prognosis, such as localization at the temple or ear [19], immunosuppression [20], or tumor budding [17,21]. These criteria need to be discussed in addition to the stratification of patients by classification, further complicating the selection of “high-risk” patients. 

In addition to consolidating the findings of Ruiz et al. [6] and Roscher et al. [13], our study represents the first attempt to compare the BWH classification with the UICC8 and NCCN classifications. It is worth noting that the four staging classifications evaluated in this study were developed to guide the management of patients in real-world scenarios. Therefore, it is crucial to compare these classifications using data from patients in real-life settings, as we have done in our study, rather than relying solely on data from highly selected patients involved in clinical trials. Based on our results, the BWH classification demonstrates superior performance in predicting the risk of poor outcomes in patients with HNCSCC when compared to the AJCC8, UICC8, and NCCN classifications. Consequently, it should be adopted to identify “high-risk” patients in clinical practice.

Further studies can be conducted to enhance the performance of the BWH classification. Currently, the classification exhibits limited reproducibility and a relatively low positive predictive value (PPV), particularly for predicting the risk of lymph node recurrence (13%). However, most recommended or evaluated treatments aim to mitigate the risk of nodal recurrence. Therefore, there is a potential risk of subjecting patients to unnecessary treatments, such as radiotherapy, which may result in morbidity if administered to poorly selected individuals. To improve the selection of these patients, some authors have proposed replacing existing prognostic classifications with molecular biomarkers. In this regard, Wysong et al. [22] reported the validation of a gene expression profile in patients at risk of lymph node metastasis, exceeding the predictive capabilities of the BWH, AJCC8, and NCCN classifications [22] and allowing for the selection of “high-risk” tumors based on biopsy before resection, to perform the appropriate surgical treatment. Additionally, it has been shown that tissue expressions of certain biomarkers, such as Cytokeratin [21], Podoplanin, Vimentin, Programmed Cell Death Ligand-1, or Epidermal Growth Factor Receptor, are significantly associated with the risk of lymph node metastasis [23,24,25]. These biomarkers could be used to create a prognostic immuno-histochemical test which could, when combined with the BWH classification, significantly increase its performance. 

## 5. Conclusions

This study is the first to compare the BWH classification to the UICC8 and NCCN classifications. It suggests that the BWH classification is the most appropriate for predicting the risk of poor outcomes in patients with HNCSCC when compared with the NCCN, UICC8, and AJCC8 classifications. However, the identification of molecular prognostic biomarkers could enhance the BWH classification by improving its reproducibility and its performance. 

## Figures and Tables

**Table 1:** High-risk status according to the 4 prognostic classifications.

Classification	High-Risk Status
BWH	>1 high-risk factor (tumor ≥2 cm in greatest diameter, poorly differentiated histology, perineural invasion of nerves >0.1 mm in caliber, tumor invasion beyond subcutaneous fat)
AJCC8	Tumor ≥4 cm in greatest diameter, or deep invasion (beyond the subcutaneous fat or >6 mm, as measured from the granular layer of adjacent normal epidermis to the base of the tumor), or bone invasion, or perineural invasion (tumor cells in the nerve sheath of a nerve lying deeper than the dermis, or measured 0.1 mm, or presenting with clinical or radiographic involvement of named nerves)
UICC8	Tumor >4 cm in greatest diameter, or deep invasion (beyond the subcutaneous fat or >6 mm, as measured from the granular layer of the adjacent normal epidermis to the base of the tumor), or bone invasion, or perineural invasion (defined as clinical or radiographic involvement of named nerves)
NCCN	Size ≥1 cm in M area, location in H area, poorly defined border, recurrent tumor, immunosuppression, site of prior radiotherapy or chronic inflammatory process, rapidly growing tumor, neurologic symptoms, poorly differentiated tumor, subtypes (acantholytic, adenosquamous, desmoplastic or metaplastic), deep invasion (beyond the subcutaneous fat or >6 mm, as measured from the granular layer of the adjacent normal epidermis to the base of the tumor), perineural or lymphatic or vascular involvement.

Abbreviations: BWH: Brigham and Women’s Hospital, AJCC8: American Joint Committee on Cancer 8th edition, UICC8: Union for International Cancer Control 8th edition, NCCN: National Comprehensive Cancer Network version 2.2020, M area: cheek, forehead, scalp and neck, H area: central face, eyelids, eyebrows, periorbital, nose, lips, chin, mandible, preauricular and postauricular skin, temple, and ear.

**Table 2:** Patient and tumor characteristics.

** Patient Characteristics (*n* = 160)**	
Age at surgery, mean [SD]	79.7 [10.1]
Sex, No (%)	
Male/Women	135 (84)/25 (16)
Follow-up time in months, median [IQR]	20 [7–48]
** Tumor characteristics (*n* = 217)**	
Immunosuppression context, No (%)	
No/Yes	177 (82)/40 (18)
Location, No (%)	
Head and neck (not ear or temple)/Ear or temple	160 (74)/57 (26)
Surgery, No (%)	
Wide excision/MOHS	189 (87)/28 (13)
Diameter, No (%)	
<2 cm/≥2 cm	120 (55)/97 (45)
Differentiation, No (%)	
Well or moderately differentiated/Poorly differentiated	197 (91)/20 (9)
Invasion > 6 mm ^a^, No (%)	
no/yes	105 (48)/53 (25)
Unknown	59 (27)
Histological perineural invasion, No (%)	
No/Yes	186 (86)/31 (14)
Tumor depth, No (%)	
Dermis/subcutaneous fat/Beyond subcutaneous fat	163 (75)/54 (25)
Risk group according to BWH, No (%)	
Low-risk/High-risk	163 (75)/54 (25)
Risk group according to UICC8, No (%)	
Low-risk/High-risk	128 (59)/89 (41)
Risk group according to AJCC8, No (%)	
Low-risk/High-risk	117 (54)/100 (46)
Risk group according to NCCN, No (%)	
Low-risk/High-risk	7 (3)/210 (97)

Abbreviations: AJCC8: American Joint Committee on Cancer 8th edition, UICC8: Union for International Cancer Control 8th edition, BWH: Brigham and Women’s Hospital, NCCN: National Comprehensive Cancer Network. ^a^ As measured from the granular layer of the adjacent normal epidermis to the base of the tumor.

**Table 3:** Risk of LR, NR, and SD.

Classification	No. (%)	LR		NR		DSD	
		No.	HR (95% CI, *p* Value ^a^)	No.	HR (95% CI, *p* Value ^a^)	No.	HR (95% CI, *p* Value ^a^)
**BWH**							
Low-risk	163 (75)	9		4		5	
High-risk	54 (25)	12	4.44 (1.87–10.55, *p* = 0.001)	7	5.48 (1.60–18.75, *p* = 0.007)	4	3.61 (0.90–14.58, *p* = 0.07)
**AJCC8**							
Low-risk	117 (54)	4		1		2	
High-risk	100 (46)	17	5.35 (1.80–15.92, *p* = 0.003)	10	12.26 (1.57–95.81, *p* = 0.02)	7	4.07 (0.84–19.75, *p* = 0.08)
**UICC8**							
Low-risk	128 (59)	4		2		2	
High-risk	89 (41)	17	6.69 (2.25–19.89, *p* = 0.001)	9	6.77 (1.46–31.34, *p* = 0.02)	7	4.96 (1.02–24.13, *p* = 0.05)
**NCCN**							
Low-risk	7 (3)	0		0		0	
High-risk	210 (97)	21	NA ^b^	11	NA ^b^	9	NA ^b^

Abbreviations: LR: local recurrence, NR: nodal recurrence, DSD: disease-specific death, AJCC8: American Joint Committee on Cancer 8th edition, UICC8: Union for International Cancer Control 8th edition, BWH: Brigham and Women’s Hospital, NCCN: National Comprehensive Cancer Network. ^a^ *p*-value based on Cox-proportional hazards regression ^b^ HR could not be evaluated because the number of “low-risk” and “high-risk” tumors according to this classification were not comparable.

**Table 4:** Evaluation of AJCC8, UICC8, BWH, and NCCN homogeneity and monotonicity.

Classification	No/Total No (%)		
	LR	NR	DSD
**Homogeneity**			
**BWH vs. AJCC 8**			
BWH low-risk	9/21 (43)	4/11 (36)	5/9 (56)
AJCC 8 low-risk	4/21 (19)	1/11 (9)	2/9 (22)
*p-*value ^a^	0.07	0.25	0.25
**BWH vs. UICC8**			
BWH low-risk	9/21 (43)	4/11 (36)	5/9 (56)
UICC8 low-risk	4/21 (19)	2/11 (18)	2/9 (22)
*p*-value ^a^	0.07	0.62	0.25
**BWH vs. NCCN**			
BWH low-risk	9/21 (43)	4/11 (36)	5/9 (56)
NCCN low-risk	0/21 (0)	0/11 (0)	0/9 (0)
*p*-value ^a^	0.008	0.13	0.07
**Monotonicity**			
**BWH vs. AJCC 8**			
BWH high-risk	12/21 (57)	7/11 (64)	4/9 (44)
AJCC 8 high-risk	17/21 (81)	10/11 (91)	7/9 (78)
*p-*value ^a^	0.07	0.25	0.25
**BWH vs. UICC8**			
BWH high-risk	12/21 (57)	7/11 (64)	4/9 (44)
UICC8 high-risk	17/21 (81)	9/11 (82)	7/9 (78)
*p-*value ^a^	0.07	0.62	0.25
**BWH vs. NCCN**			
BWH high-risk	12/21 (57)	7/11 (64)	4/9 (44)
NCCN high-risk	21/21 (100)	11/11 (100)	9/9 (100)
*p-*value ^a^	0.008	0.13	0.07

Abbreviations: LR: local recurrence, NR: nodal recurrence, DSD: disease-specific death, AJCC8: American Joint Committee on Cancer 8th edition, UICC8: Union for International Cancer Control 8th edition, BWH: Brigham and Women’s Hospital, NCCN: National Comprehensive Cancer Network. ^a^ *p*-value based on the McNemar test.

**Table 5:** Sensitivity, specificity, PPV, and NPV of BWH, AJCC8, UICC8, and NCCN high-risk tumors to predict poor outcomes (LR, NR, and DSD considered a single risk) and to predict NR.

Classification	Sensitivity [95% CI]	Specificity [95% CI]	PPV [95% CI]	NPV [95% CI]	C–Index
To Predict LR, NR, and DSD (Combined as a Single Risk)					
**BWH vs. AJCC 8**					
BWH	0.55 [0.36–0.74]	0.80 [0.73–0.85]	0.30 [0.18–0.44]	0.92 [0.87–0.96]	0.68
AJCC 8	0.83 [0.64–0.94]	0.60 [0.52–0.67]	0.24 [0.16–0.34]	0.96 [0.9–0.99]	0.71
*p*-value	0.01 ^a^	<0.001 ^a^	NA ^c^	NA ^c^	0.41 ^b^
**BWH vs. UICC8**					
BWH	0.55 [0.36–0.74]	0.80 [0.73–0.85]	0.30 [0.18–0.44]	0.92 [0.87–0.96]	0.68
UICC8	0.79 [0.6–0.92]	0.65 [0.58–0.72]	0.26 [0.17–0.36]	0.95 [0.9–0.98]	0.72
*p*-value	0.05 ^a^	<0.001 ^a^	NA ^c^	NA ^c^	0.36 ^b^
**BWH vs. NCCN**					
BWH	0.55 [0.36–0.74]	0.80 [0.73–0.85]	0.30 [0.18–0.44]	0.92 [0.87–0.96]	0.68
NCCN	1 [0.88–1]	0.04 [0.02–0.07]	0.14 [0.09–0.19]	1 [0.59–1]	0.52
*p*-value	0.001 ^a^	<0.001 ^a^	NA ^c^	NA ^c^	0.002 ^b^
To predict NR					
**BWH vs. AJCC 8**					
BWH	0.64 [0.31–0.89]	0.77 [0.71–0.83]	0.13 [0.05–0.25]	0.98 [0.94–0.99]	0.70
AJCC 8	0.91 [0.59–1]	0.56 [0.49–0.63]	0.10 [0.05–0.18]	0.99 [0.95–1]	0.74
*p*-value	0.25 ^a^	<0.001 ^a^	NA ^c^	NA ^c^	0.66 ^b^
**BWH vs. UICC8**					
BWH	0.64 [0.31–0.89]	0.77 [0.71–0.83]	0.13 [0.05–0.25]	0.98 [0.94–0.99]	0.70
UICC8	0.82 [0.48–0.98]	0.61 [0.54–0.68]	0.10 [0.05–0.18]	0.98 [0.94–1]	0.71
*p*-value	0.62 ^a^	<0.001 ^a^	NA ^c^	NA ^c^	0.91 ^b^
**BWH vs. NCCN**					
BWH	0.64 [0.31–0.89]	0.77 [0.71–0.83]	0.13 [0.05–0.25]	0.98 [0.94–0.99]	0.70
NCCN	1 [0.72–1]	0.03 [0.01–0.07]	0.05 [0.03–0.09]	1 [0.59–1]	0.52
*p*-value	0.13 ^a^	<0.001 ^a^	NA ^c^	NA ^c^	0.02 ^b^

Abbreviations: LR: local recurrence, NR: nodal recurrence, DSD: disease-specific death, AJCC8: American Joint Committee on Cancer 8th edition, UICC8: Union for International Cancer Control 8th edition, BWH: Brigham and Women’s Hospital, NCCN: National Comprehensive Cancer Network, PPV: positive predictive value, NPV: negative predictive value ^a^ *p*-value based on the McNemar test ^b^ *p*-value based on the Delong test ^c^ *p*-value cannot be estimated for positive and negative predictive values because they are based on the prevalence of the disease.

## Data Availability

The data used for this study is not made available to the public.
